# Megakaryocytes promote osteoclastogenesis in aging

**DOI:** 10.18632/aging.103595

**Published:** 2020-07-07

**Authors:** Deepa Kanagasabapathy, Rachel J. Blosser, Kevin A. Maupin, Jung Min Hong, Marta Alvarez, Joydeep Ghosh, Safa F. Mohamad, Alexandra Aguilar-Perez, Edward F. Srour, Melissa A. Kacena, Angela Bruzzaniti

**Affiliations:** 1 Department of Orthopaedic Surgery, Indiana University School of Medicine, Indianapolis, IN 46202, USA; 2 Department of Biomedical Sciences and Comprehensive Care, Indiana University School of Dentistry, Indianapolis, IN 46202, USA; 3 Department of Medicine, Indiana University School of Medicine, Indianapolis, IN 46202, USA

**Keywords:** megakaryocyte, osteoclast, bone marrow macrophage, aging, thrombopoietin

## Abstract

Megakaryocytes (MKs) support bone formation by stimulating osteoblasts (OBs) and inhibiting osteoclasts (OCs). Aging results in higher bone resorption, leading to bone loss. Whereas previous studies showed the effects of aging on MK-mediated bone formation, the effects of aging on MK-mediated OC formation is poorly understood. Here we examined the effect of thrombopoietin (TPO) and MK-derived conditioned media (CM) from young (3-4 months) and aged (22-25 months) mice on OC precursors. Our findings showed that aging significantly increased OC formation in vitro. Moreover, the expression of the TPO receptor, Mpl, and circulating TPO levels were elevated in the bone marrow cavity. We previously showed that MKs from young mice secrete factors that inhibit OC differentiation. However, rather than inhibiting OC development, we found that MKs from aged mice promote OC formation. Interestingly, these age-related changes in MK functionality were only observed using female MKs, potentially implicating the sex steroid, estrogen, in signaling. Further, RANKL expression was highly elevated in aged MKs suggesting MK-derived RANKL signaling may promote osteoclastogenesis in aging. Taken together, these data suggest that modulation in TPO-Mpl expression in bone marrow and age-related changes in the MK secretome promote osteoclastogenesis to impact skeletal aging.

## INTRODUCTION

Bone remodeling is a continuous and highly synchronized process which occurs throughout the lifetime of an individual. As aging progresses, the activities of the bone cells, including osteoclasts (OCs) and osteoblasts (OBs) become dysregulated, with osteoclast activity exceeding osteoblastic bone formation, leading to bone loss. Although both females and males experience osteoporosis in aging, post-menopausal women suffer higher bone resorption levels due to low estrogen levels [1–3]. While many risk factors are associated with declining bone mass in aging, a complete understanding of the cellular mechanisms related to bone loss and the impact of these cells within the bone marrow is still lacking.

A growing body of evidence suggests that megakaryocytes (MKs), which are platelet-producing cells, regulate the skeletal system by impacting the growth and function of both the mesenchymal-derived OBs and hematopoietic-derived OCs [4–15]. Many human conditions as wells as animal models demonstrate a strong link between high MK numbers and high bone mass [6, 16–20]. Mice with elevated MK numbers exhibit up to a 3-fold increase in bone mass in vivo, which is attributed in part to MK-stimulated OB proliferation [6, 11, 15, 20]. However, the MK-mediated stimulation of OB proliferation and bone formation is impaired with aging, despite the marked increase in MK number in vivo, suggesting that aged MKs have decreased capacity to stimulate bone formation [21].

In contrast to stimulating OB activity, MKs are known to negatively impact OC differentiation. MKs from human cord blood or murine fetal livers significantly inhibit OC formation in vitro [5, 11, 14]. However, the mechanisms and secreted factors that control the effects of MKs on osteoclastogenesis are not entirely clear. MKs are also known to express estrogen receptors, and treatment with estrogen impacts MK function, including the secretion of OPG and RANKL, which are known to regulate OC number [4, 19, 22], but whether these factors are changed with aging, resulting in increased OC formation is unknown. Therefore, in the current study, we investigated the influence of aging on MK-regulated OC formation.

## RESULTS

### Increased osteoclastogenesis by CD45+F4/80+ macrophages from aged mice

**Figure 1:**
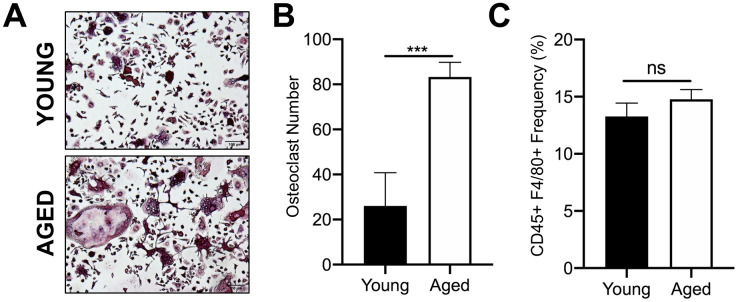
**Aging increases OC formation from sorted CD45+F4/80+ bone marrow macrophages.** Similar numbers of sorted CD45+F4/80+ macrophages from young and aged C57BL/6J mice were cultured in the presence of RANKL (80 ng/ml) and M-CSF (20 ng/ml). Growth media was changed every second day for 5-7 days until mature, multinucleated OCs (>3 nuclei) were formed. (**A**) Images of TRAP+ OCs generated. Scale bar is 100 μm. (**B**) The number of TRAP+ OCs formed in each well was quantified, showing that more OCs were generated from aged CD45+F4/80+ macrophages compared to young macrophages. Four independent experiments gave similar results, and a representative experiment is shown. Graphs are mean ± SD with ***p<0.001 (N=4/group). (**C**) Frequency of CD45+F4/80+ macrophage (%) in bone marrow cells isolated from young and aged C57BL/6J mice (males and females combined). Data are mean ± SD of four independent experiments (*p*=0.08, ns indicates non-significant).

We first confirmed that OC progenitors from aged C57BL/6J mice have increased potential to undergo osteoclastogenesis compared to those from young mice. For these studies, we used CD45+F4/80+ macrophages isolated by fluorescence activated cell sorting (FACS) of bone marrow from aged (22-25 months) and young (3-4 months) mice. We previously reported that this subpopulation are also CD11b+, which is a classical osteoclast marker [23]. In vitro osteoclastogenesis was performed by culturing these cells with the necessary OC cytokines, RANKL (80 ng/ml) and M-CSF (20 ng/ml). Similar to previous reports [24], the number of TRAP+ multinucleated osteoclasts generated from aged CD45+F4/80+ macrophages was 3.2-fold higher than that generated from young CD45+F4/80+ macrophages (***p<0.001) (Figure 1A, 1B). However, aging did not appear to affect the frequency of the CD45+F4/80+ macrophage subpopulation in the bone marrow of young versus aged mice (Figure 1C).

### Megakaryocytes and TPO are increased in aged bone marrow

**Figure 2:**
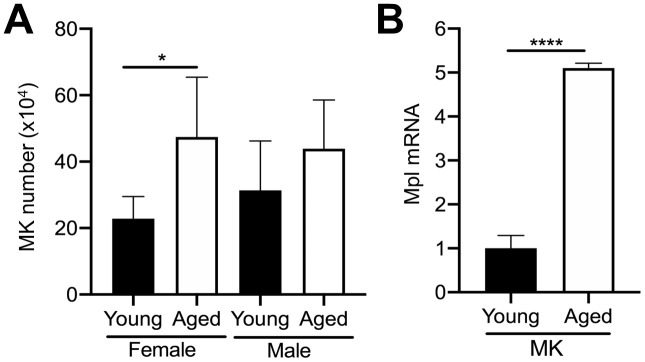
**Aging increases bone marrow MK number.** (**A**) Flow cytometry was used to determine the total number of CD45+CD41+ MKs in the bone marrow of young and aged C57BL/6J female and male mice. The data are presented as mean ± SD of four independent experiments with *p<0.05 (N=4/group). (**B**) Bone marrow CD45+CD41+ MKs were isolated by FACS from young and aged mice (males and females were combined) and used for real-time PCR analysis of relative Mpl mRNA expression between groups. The Ct cycle range was 28.99–33.83. The data are presented as mean ± SD of three independent experiment (****p<0.0001; N=3-6/group).

Given that MKs occupy a strategic position within the bone marrow cavity and are known to regulate OC formation [11, 14, 19], we examined if aging is correlated with changes in the number of bone marrow MKs in male and female mice. We compared the total number of MKs (CD45+CD41+ cells) in the bone marrow of aged and young C57BL/6J mice by flow cytometry. Interestingly, we found approximately a 2.1-fold increase in the number of MKs in flushed bone marrow from female aged mice, compared to female young mice (Figure 2A). A trend towards increased MK number in male aged mice was also observed but did not reach statistical significance.

MK proliferation and differentiation are regulated by the growth factor, thrombopoietin (TPO), which acts via its specific receptor Mpl [16, 25, 26]. Therefore, we examined Mpl expression in MKs from aged versus young mice. Interestingly, aged MKs express significantly higher Mpl levels than young MKs (Figure 2B). Together with the increase in bone marrow MK numbers in aged mice (Figure 2A), these findings suggest that the increase in MK number with aging may be the result of increased megakaryopoiesis, which is supported by our previous studies [21].

**Figure 3:**
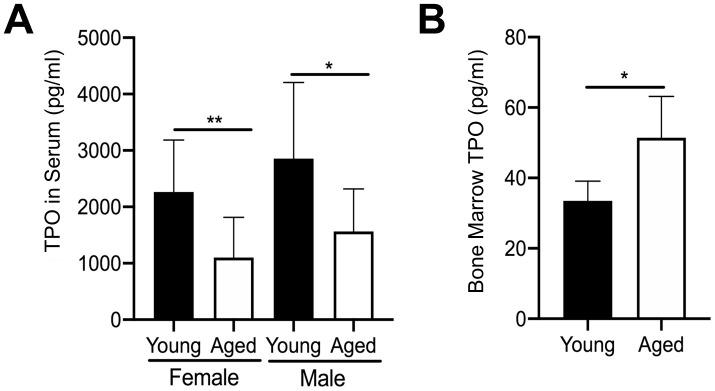
**Changes in TPO levels in young and aged mice.** (**A**) TPO concentration was measured by ELISA in serum from young and aged, male and female C57BL/6J mice. Serum TPO was significantly reduced in aged female and male mice compared to sex-matched young mice. The data are presented as mean ± SD (*p<0.05, **p<0.01, N=9-10/group). (**B**) Bone marrow supernatant was collected from young and aged femur and tibia (male and female mice were combined) and used to determine TPO concentrations by ELISA. The data are presented as mean ± SD (*p<0.05; N=5/group).

We further examined the mechanism leading to the increase in MK number in aged male and female mice by quantifying extracellular TPO levels. We first quantified TPO in serum from aged versus young mice using a specific ELISA. In addition, given that bone marrow MKs are likely to be influenced by changes in local paracrine factors, we also isolated bone marrow supernatant from hindlimbs of young and aged mice. However, due to the small amount of bone marrow supernatant recovered, male and female mice were combined. As shown in Figure 3A, TPO was significantly lower in the serum of aged mice versus young mice for both female and male cohorts. In contrast, TPO levels in bone marrow supernatant were significantly higher in aged mice compared to young mice (Figure 3B). These findings may suggest that increased TPO in the bone marrow, combined with increased Mpl expression in MKs, may be driving a localized increase in MK numbers with aging.

### Direct effects of TPO on osteoclastogenesis

**Figure 4:**
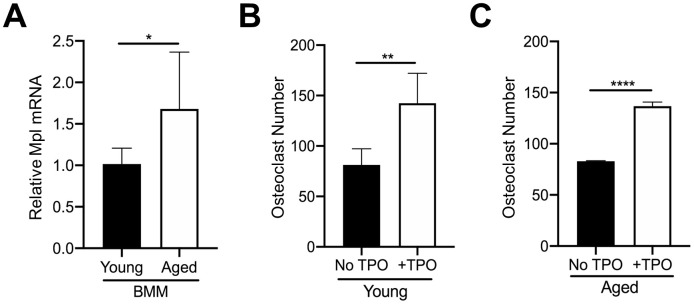
**TPO stimulates OC formation.** (**A**) FACS sorted CD45+F4/80+ macrophages from young and aged mice were subject to real-time PCR to assess the relative mRNA expression of Mpl. The Ct cycle range was 34.06–35.15. The data shown are mean ± SD (*p<0.05). (**B**, **C**) FACS sorted CD45+F4/80+ macrophages were cultured with 20 ng/ml M-CSF and 80 ng/ml RANKL in the presence or absence of recombinant human TPO (100 ng/ml). Growth media was changed every second day for 5–7 days until mature, multinucleated OCs (>3 nuclei) were formed. Mature OCs were fixed and TRAP+ OCs were counted. The data are presented as mean ± SD of four (female) and two (male) independent experiments (*p<0.05, ***p<0.001; N=3/group).

In addition to MKs, Mpl is expressed on several other cell types within the bone marrow. For example, our previous findings confirmed that OC progenitors from 6-8 week-old C57BL/6J mice express Mpl, and robustly respond to recombinant TPO, resulting in an increase in OC formation [27, 28]. Therefore, we examined if osteoclastogenesis associated with aging is affected by differences in Mpl-TPO signaling. As Mpl is expressed on several cell types within the bone marrow cavity we isolated CD45+F4/80+ macrophages by FACS from young and aged mice, and analyzed for Mpl expression by real-time quantitative PCR. As shown in Figure 4A, CD45+F4/80+ macrophages from aged mice express significantly higher Mpl mRNA than young CD45+F4/80+ macrophages, suggesting they may be more responsive to TPO. To test this, we examined possible differential responses to TPO-induced osteoclastogenesis. CD45+F4/80+ macrophages were isolated from the bone marrow of young and aged mice and then cultured in the presence of M-CSF and RANKL, supplemented without or with 100 ng/ml TPO. The concentration of TPO used was previously demonstrated to induce osteoclastogenesis in macrophage cultures [28]. As shown in Figures 4B, 4C, TPO stimulated a significant increase in OC number in both young and aged CD45+F4/80+ macrophages, suggesting that TPO-induced osteoclastogenesis is independent of aging.

### Aging impairs the ability of megakaryocytes to inhibit osteoclast differentiation

The studies above revealed an increase in bone marrow TPO levels in aged mice compared to young mice, and that TPO increases OC formation in CD45+F4/80+ macrophages isolated from both young and aged mice (Figures 3, 4). Moreover, we observed an increase in MK number in aged bone marrow (Figure 2). However, we and others also showed that MKs derived from fetal livers (males and females were not separated) or cord blood secrete factors that inhibit OC formation [11, 14, 19]. To investigate this apparent contradiction, we examined whether MKs from aged mice have altered functionality with respect to osteoclastogenesis.

**Figure 5:**
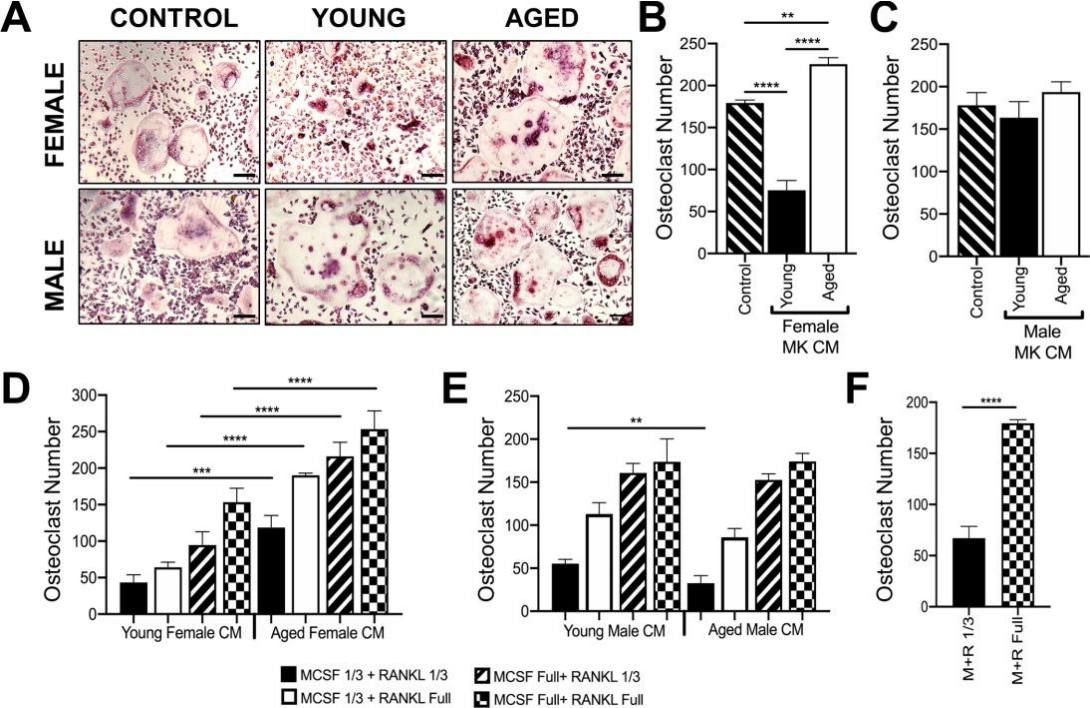
**Effect of MK conditioned media on OC formation.** (**A**–**E**) Conditioned medium (CM) was prepared from MKs isolated from young and aged, female and male mice. (**A**–**C**) Unsorted BMMs from mice (6-10 weeks) were cultured in media containing 25% (v/v) MK CM plus RANKL (80 ng/ml) and M-CSF (20 ng/ml). The control media was prepared without MKs. (**A**) Micrographs showing mature OCs. Scale bar is 50 μm. (**B**, **C**) The number of TRAP+ multinucleated OC (>3 nuclei) was quantified. The data show that MK CM from young female mice inhibits OC formation compared to control and aged female MK CM. The data are presented as mean ± SD of three experiments (****p<0.0001 compared to the control group). No significant difference in OC formation was observed between male groups. (**D**, **E**) Unsorted BMMs from mice (6-10 weeks) were differentiated into mature OCs in media containing 25% MK CM plus normal or reduced RANKL (80 or 26.6 ng/ml, respectively) and M-CSF (6.6 or 20 ng/ml, respectively). Mature OCs were fixed and TRAP+ OCs were counted. (**F**) Comparison of OCs formed in control media (no MK CM) with normal or reduced RANKL and M-CSF. Three independent experiments showed similar results and a representative figure is shown. The data are presented as mean ± SD (**p<0.01, ***p<0.001, ****p<0.0001; N= 3/group). P-values were calculated by one-way ANOVA followed by Tukey’s post-hoc test.

MKs were isolated from young and aged, male and female mice, and used to prepare conditioned media (CM) as described in the methods [14]. Unsorted bone marrow macrophages or BMMs (OC-progenitors) from the bone marrow of 6-10 week-old mice were then cultured in media containing 25% (v/v) MK CM plus RANKL (80 ng/ml) and M-CSF (20 ng/ml). Control media containing only RANKL and M-CSF was also used. Figures 5A, 5B show that OC formation was lower in cultures containing CM from female young MKs compared to control media, confirming that MKs inhibit osteoclastogenesis as we reported previously [14]. In contrast, cultures containing female aged MK CM produced significantly more OCs than young female MK CM (*p*<0.0001) or control media (*p*<0.01). On the other hand, as illustrated in Figure 5C, male MK CM showed no significant effect on OC formation whether or not MKs were derived from young or aged mice (*p*>0.05).

Since RANKL and M-CSF are required for in vitro osteoclastogenesis assays, we also conducted studies in media containing 25% (v/v) MK CM plus one-third reduced concentrations of RANKL and/or M-CSF (Figure 5D, 5E), which gave similar findings. As expected, a reduction in overall OC numbers was observed in culture media (without CM) using the sub-optimal RANKL and M-CSF conditions as shown in Figure 5F.

### Modulation of RANKL in megakaryocytes during aging

**Figure 6:**
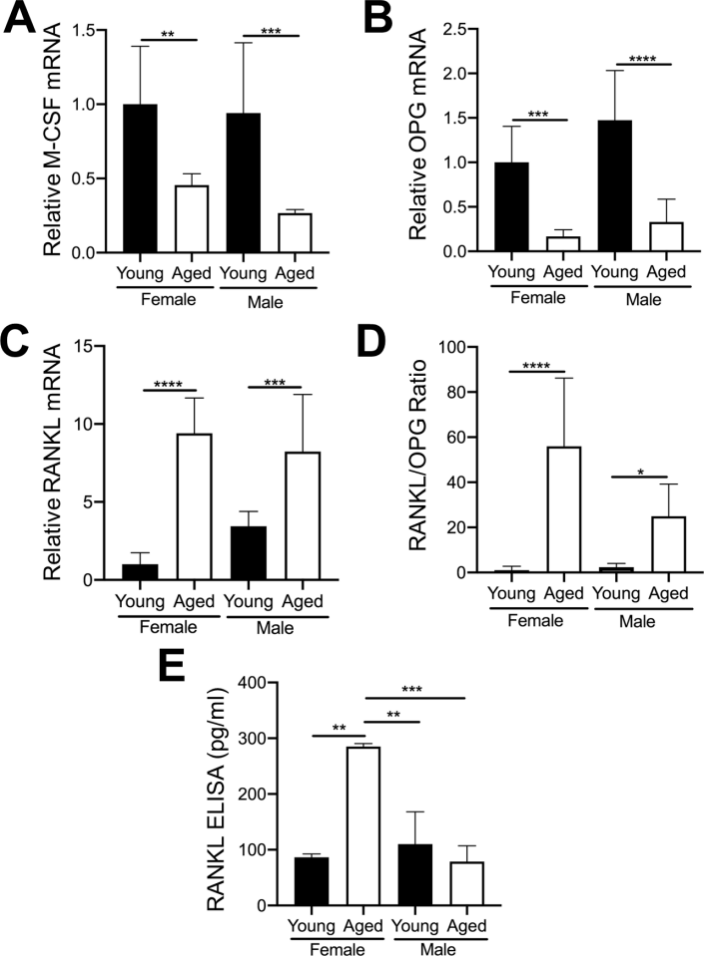
**Expression of key OC factors in MKs from young and aged mice.** (**A**–**D**) MKs were prepared from the bone marrow of young and aged, male and female mice using a BSA gradient and used for mRNA expression analyses by real-time PCR. (**A**) M-CSF mRNA (Ct cycle range was 25.2–30.12). (**B**) OPG mRNA (Ct range 35.09–39.53). (**C**) RANKL mRNA (Ct range 28.6–35.5). (**D**) The RANKL/OPG ratio was calculated for young and aged, male and female MKs (N=4-5/group). (**E**) RANKL concentrations in MK CM was quantified by ELISA. MK CM from aged females was significantly higher than MK CM from young females. *P*-values were calculated by one-way ANOVA followed by Tukey’s post-hoc test and the data are presented as mean ± SD. (*p<0.05, **p<0.01, ***p<0.001, ****p<0.0001).

The importance of RANKL and M-CSF for osteoclastogenesis is well established [29, 30]. OC numbers are also affected by osteoprotegerin (OPG) which blocks the ability of RANKL to bind to the RANK receptor on OC precursors [31]. Furthermore, as aging progresses, the level of RANKL in whole bone and in cultured bone marrow cells from both humans and rodents gradually increases [32]. Therefore, we examined whether MKs from young and aged mice express different mRNA levels of these cytokines. As shown in Figure 6A, 6B, MKs from aged female or male mice showed reduced expression of M-CSF (Figure 6A) and OPG (Figure 6B) compared to sex-matched young mice. However, RANKL mRNA (Figure 6C) was markedly higher in MKs from aged mice compared to young mice, for both females and males. Moreover, the ratio of RANKL/OPG (Figure 6D) was increased in MKs from aged mice, suggesting increased potential to induce osteoclastogenesis compared to MKs from young mice. We next confirmed whether MKs secrete RANKL, as determined by specific ELISA. Consistent with an increase in RANKL mRNA, we observed that MKs from aged female mice secrete RANKL at significantly higher levels than MKs from young female mice, young male mice, as well as aged male mice (Figure 6E). Together, these data suggest that MKs from aged female mice may stimulate OC formation through increased RANKL signaling.

## DISCUSSION

Increases in OC activity result in bone loss as is seen with menopause and aging. MKs can regulate bone mass by regulating bone formation as well as bone resorption. In several mouse models with increased bone marrow megakaryopoiesis, a significant increase in bone volume, due to increases in bone formation, has been reported; adult mice less than 9 months of age were used for these studies [6, 15, 20, 21, 33–37]. Our group [14] and others [5] have also shown that MKs from young mice or human umbilical cord blood, respectively, secrete factor(s) that inhibit OC formation up to 10-fold. These studies suggest that in young mice, MKs contribute to bone gain through both increased bone formation and decreased osteoclast resorption. However, whether this is different between males and females has not been investigated. Moreover, the signaling factors secreted by young MKs versus aged MKs that differentially regulate OC formation during aging remain to be identified. As it has become increasingly clear that aging results in the dysregulation of a number of cells and systems, we explored how aging affects the ability of MKs to inhibit OC formation and the role of TPO, the principal MK-growth factor, on OC formation.

In the current study, we found that MK CM from young female mice, but not young male mice inhibited OC formation (Figure 5). In contrast, MK CM from aged female mice led to an increase in OC formation. Although in the current study we did not investigate direct coculture of aged MKs with OC progenitors, we previously reported that OC formation is inhibited by both direct contact with young female MKs and by young MK CM [14], and we therefore expect similar findings using aged MKs. The increase in MK-mediated OC formation in aging may be due to an increase in pro-osteoclastic factors, including RANKL. We found that RANKL mRNA expression was markedly elevated in aged MKs from both male and female mice (Figure 6C). However, only CM from aged female MKs showed significantly elevated RANKL protein levels, compared with sex-matched young mice. These data not only suggest that MKs from aged female mice may stimulate OC formation through increased RANKL secretion, but that RANKL mRNA translation, secretion, or protein stability may be different in aged male MKs versus aged female MKs. Together, our data indicate sexual dimorphism in the regulation of RANKL and the age-associated osteoclastogenic effects of MKs.

In post-menopausal women treated with estrogen, a correlation between increased MK numbers and high bone mass has been previously reported [19, 22]. Further, estradiol treatment of CD61+ MK lineage cells isolated from human cord blood cells was reported to significantly increase OPG expression, and simultaneously reduce RANKL expression [38]. Together with our current findings, it is possible that in conditions of reduced estrogen levels as occurs in aging, MKs may express high RANKL levels, which would support increased osteoclastogenesis. These finding are important since denosumab, an anti-RANKL therapy, is currently used for the treatment of osteoporosis [39], and our findings suggest that MKs are an important cellular source of RANKL that may contribute to bone loss in aging.

An alternative mechanism by which aged MKs increase OC formation by could be the loss of a yet-to-be determined inhibitory factor(s). We previously published that OC formation was similar between MKs from young OPG-deficient mice and young wild-type mice [14]. Based on these previous findings, it is unlikely that OPG is responsible for the inhibition of OC formation observed by MKs from young female mice in the current study (Figure 5). However, the decline in secreted OPG in aged MKs may, contribute to the increase in OC formation by increasing the RANKL/OPG ratio as we observed (Figure 6).

Bone loss in aging is generally associated with an overall increase in the number of bone resorbing OCs. This could be due to an increase in the number of OC progenitors such as BMMs, or increased proliferation of BMMs. Accelerated osteoclastogenesis or increased/decreased expression of OC-regulating factors within the bone marrow environment may also be involved. Our studies reveal that sorted CD45+F4/80+ macrophages (OC progenitors) from aged mice show increased capacity to undergo osteoclastogenesis, compared with those from young mice (Figure 1). We also found that aging increases the number of MKs within the bone marrow cavity (Figure 2). The increase in both MK numbers and OC formation with aging may be influenced by hematopoietic skewing to the myeloid lineage, which is known to occur with aging [40, 41]. It was reported that aged human OC progenitors express higher RANK mRNA, which may make them more susceptible to RANKL stimulation [42]. Other groups have also shown that RANKL levels in whole bone and cultured bone marrow cells gradually increase with age in both humans and rodents [43, 44]. Combined with these reports, our findings that aging increases MK-derived RANKL expression and secretion (Figure 6) suggest that increased RANKL signaling is likely a key driver of the increase in OC formation by MKs in aging.

As TPO is the main growth factor for MKs, we examined TPO levels in sera and bone marrow supernatant of young and aged mice (Figure 3). An unexpected reduction in serum TPO was observed in aged mice compared to young controls. Although it is currently unclear why systemic TPO levels decline with aging, some possibilities may include an age-associated decline in TPO production by primary (e.g., liver) or secondary (e.g., kidney) tissue sources, or that TPO is sequestered away from the circulation. Alternatively, the reduction in serum TPO could be explained by the higher numbers of platelets [21], which can bind circulating TPO, a well-documented phenomenon [45]. Despite the reduction in circulating TPO, we found increased TPO concentrations in the bone marrow supernatant of aged mice compared to young mice (Figure 3). As demonstrated here and in our previous publications [28], recombinant TPO (without the presence of MKs) increased the number of mature TRAP+ multinucleated OCs generated from young and aged OC progenitors. By contrast, Wakikawa et al. [46] used whole bone marrow cultures containing both OC progenitors and MKs/MK progenitors and found that TPO inhibited OC development, likely owing to the MK-mediated inhibition of OC formation. Our current studies also confirm that the TPO receptor, Mpl (also known as CD110), is more highly expressed on OC progenitors from aged mice, compared to young mice (Figure 4A). Further, we reported that Mpl is required for TPO to enhance OC formation in vitro [28]. However, it is unknown whether in vivo the higher level of TPO in aged bone marrow preferentially acts on Mpl on MKs to increase MK number (Figure 2), which in turn affects OC formation, or alternatively whether TPO acts directly on OC-progenitors to increase OC formation (Figure 4).

**Figure 7:**
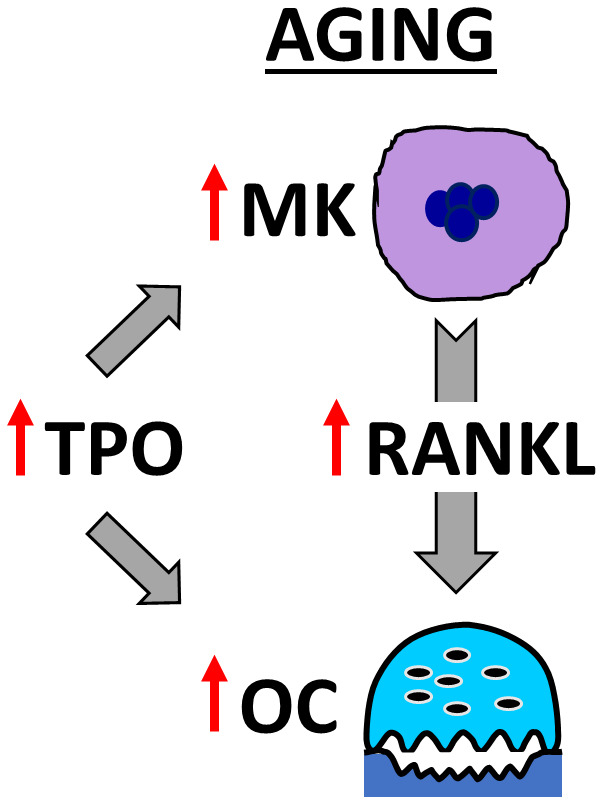
**Model showing OC stimulation by TPO and MKs in aging.** The schema illustrates that aging increases MK number and alters MK-secreted factors, including RANKL, which together promote osteoclastogenesis. In addition, elevated TPO in the bone marrow cavity promotes direct and MK-mediated effects on OC formation in aged mice.

In summary, our findings demonstrate that Mpl, the receptor for TPO [47], is upregulated in OC-progenitor cells in aging. Our studies also demonstrate that BMMs from young and aged mice respond similarly to TPO, leading to increased OC formation. Moreover, we found that aged bone marrow contains more OCs, MKs, and TPO compared to young mice; and that MKs from aged female mice secrete more RANKL than young female, young male, or aged male mice. As summarized in Figure 7, these findings support both the direct effects of TPO on OC formation, as well as TPO-driven increases in MK number and RANKL secretion to promote OC formation and decrease bone mass in aging.

## MATERIALS AND METHODS

### Experimental animals and mouse strains

Animals were maintained, bred, and handled following protocols approved by the Indiana University Institutional Animal Care and Use Committee (IACUC) and in accordance with the NIH Guidelines for the care and use of laboratory animals. In our studies, young (~3-4 month-old) C57BL/6J mice were bred and housed at the Indiana University School of Medicine. Aged (~23-25 month-old) C57BL/6J mice were provided by the National Institute of Aging of the National Institutes of Health.

### Preparation of adult bone marrow-derived megakaryocytes

Single cell suspensions of bone marrow from long bones (femur and tibiae) were obtained. Cells were cultured in Dulbecco Modified Eagle Medium (DMEM, Grand island, NY, USA) containing 5% fetal bovine serum (FBS, Hyclone, GE Healthcare Life Sciences, Pittsburgh, PA), 1000 U/ml Penicillin/Streptomycin (Invitrogen, Waltham, MA), and supplemented with 1% (v/v) media collected from a murine TPO-secreting fibroblast cell line [14, 18]. MKs were isolated after ~7–10 days using a one-step BSA gradient to separate them from lymphocytes and other cells. This results in approximately a 90–95% pure MK population [15, 48].

### Preparation of megakaryocyte conditioned media

MK CM was prepared as previously described [11, 14]. In brief, 1 x 10^6^ MKs/ml were cultured in α-MEM (Grand island, NY, USA) containing no serum and no phenol red. After three days CM was collected and stored at -80°C until use. For the current study we used 25% CM (v/v) for OC development assays as described below.

### Osteoclast differentiation assays

Femurs and tibiae were dissected from mice, the epiphyses were removed, and the marrow was flushed with a 27-gauge needle and syringe containing α-MEM with 10% FBS. Bone marrow cells were suspended into single cells and washed twice prior to use. Bone marrow cells were then prepared using previously described protocols [28]. Briefly whole bone marrow was plated into 35mm tissue culture dishes in α-MEM supplemented with 10% FBS. After 24 hours, the non-adherent cells were collected. These unsorted monocyte/myeloid progenitors, which we termed bone marrow macrophages (BMMs), were used for osteoclastogenesis assays as described below. Alternatively, bone marrow cells were FACS sorted to isolate CD45+F4/80+ macrophages, which were then used in osteoclastogenesis assays as indicated.

For osteoclastogenesis assays with MK CM, BMMs from young mice 6-10 weeks of age (males and females combined), were seeded at 0.7-1.5 x 10^5^ cells in 96-well plates and cultured in α-MEM supplemented with 10% FBS and 20 ng/ml M-CSF and 80 ng/ml RANKL (PeproTech Inc., Rocky Hill, NJ). In some studies, sub-optimal concentrations of M-CSF (6.6 ng/ml) and RANKL (26.6 ng/ml) were used. MK CM at 25% vol/vol was added to the culture media.

For osteoclastogenesis assays involving TPO, FACS sorted CD45+F4/80+ macrophages were cultured at 7–9 x 10^4^ cells in 96-well plates in α-MEM supplemented with 10% FBS and 20 ng/ml M-CSF and 80 ng/ml RANKL plus 100 ng/ml recombinant human TPO (PeproTech). For all studies, growth media was changed every second day for 5-7 days until mature, multinucleated OCs (>3 nuclei) were formed. Cells were fixed and stained for tartrate resistant acidic phosphatase (TRAP, Sigma-Aldrich, St. Louis, USA). Images were acquired at 10X magnification using the Image pro plus 7.0 software on Leica DMI4000 equipped with digital camera. All experiments were repeated at least 3 times.

### Flow cytometry and cell sorting

FACS-sorted CD45+F4/80+ macrophages were prepared as previously described [6]. Briefly murine bone marrow mononuclear cells were blocked with TruStain FcX (Bio-legend, San Diego, CA) for 15 minutes at 4°C and stained with anit-CD45 (clone: 30-F11) and anti-F4/80 (clone: BM8) (Bio-legend) antibodies for 30 minutes at 4°C. Cells were washed with wash buffer (1% FBS in PBS and 1% Pen-Strep). CD45+F4/80+ macrophages were sorted using BD FACSAria and used for osteoclastogenesis assays as indicated above. For MKs, bone marrow cells were prepared and stained as above except anti-CD41 antibody (clone: MWReg30; Bio-legend) was used instead of the F4/80 antibody. MKs were CD45+CD41+ cells. Cells were acquired using the BD LSRFortessa (BD Biosciences. San Jose, CA) and analyzed by FlowJo software (BD Biosciences).

### RNA extraction and real-time PCR

Total RNA from bone marrow derived MKs or BMMs was isolated using RNeasy Plus Micro Kit (Qiagen, Valencia, CA) and used to prepare complementary DNA (cDNA) using the SuperScript™ IV VILO™ Master Mix (ThermoFisher Scientific). mRNA expression levels for each gene were quantified by quantitative real-time PCR using the CFX96 real time system (Bio-Rad, Hercules, CA, USA) with the Power SYBR™ Green PCR Master Mix (ThermoFisher Scientific). Gene expression levels were normalized to the corresponding GAPDH gene expression. All samples were run in duplicate, and at least 3 separate biologic replicates were analyzed. The Ct cycle number for each gene is shown in the figure legends.

**Table:** 

**Target Gene**	**Sequence 5’-3’**
GAPDH	F: CGTGGGGCTGCCCAGAACAT
	R: TCTCCAGGCGGCACGTCAGA
MPL	F: CAGAAATCTGCCTGCTGTGA
	R: AGGCTTGCACACAGTTCCCTTA
M-CSF	F: ATGAGCAGGAGTATTGCCAAGG
	R: TCCATTCCCAATCATGTGGCTA
OPG	F: ACCCAGAAACTGGTCATCAGC
	R: CTGCAATACACACTCATCACT
RANKL	F:CATTTGCACACCTCACCATC
	R:TCCGTTGCTTAACGTCATGT

### Detection of RANKL in megakaryocyte conditioned media

MK CM was collected as described above, and stored in aliquots at -80°C. Undiluted CM was assayed for RANKL using a specific ELISA kit (R&D Systems, Minneapolis, MN) as per the manufacturer’s instructions.

### Measurement of TPO in serum and bone marrow supernatant

Whole blood was collected from mice at necropsy by cardiac puncture, and serum was separated and stored at -80°C until assayed. Bone marrow from the hindlimbs of mice was removed through centrifugation, and the supernatant, devoid of bone marrow cells, was collected and stored at -80°C until assayed. The serum and bone marrow supernatant samples were diluted as necessary (up to 1:4) and assayed for TPO concentration using the Mouse Thrombopoietin Quantikine ELISA kit (R&D Systems, Minneapolis, MN), following the manufacturer’s instructions.

### Statistical analysis

Graphpad prism software (Graphpad Software, Inc. La Jolla, CA, USA) was used for statistical analyses. Unless otherwise specified, an unpaired two-tailed Student’s t-test was applied for comparisons between two groups. The statistical significance of differences among groups was assessed using analysis of variance (ANOVA) with a Tukey post-hoc multiple comparisons test. All data are presented as the mean ± standard deviation (SD). *P*<0.05 was considered significant. All independent experiments were replicated at least three times.
